# Impact of prosthesis position on hearing outcomes in otosclerosis patients based on ultra-high-resolution CT

**DOI:** 10.1016/j.bjorl.2025.101688

**Published:** 2025-07-29

**Authors:** Chen Yang, Heyu Ding, Ting Zhang, Pengfei Zhao, Zhenchang Wang, Shusheng Gong, Jing Xie

**Affiliations:** a Capital Medical University, Beijing Friendship Hospital, Department of Otolaryngology-Head and Neck Surgery, Beijing, China; b Capital Medical University, Clinical Center for Hearing Loss, Beijing, China; c Capital Medical University, Beijing Friendship Hospital, Department of Radiology, Beijing, China

**Keywords:** Otosclerosis, Stapes prosthesis, Stapedotomy, U-HRCT

## Abstract

**Objectives:**

This study aimed to explore the impact of prosthesis position on hearing outcomes in otosclerosis patients based on Ultra-High-Resolution CT (U-HRCT).

**Methods:**

We retrospectively reviewed medical records of 142 patients (182 ears) who underwent stapedotomy. We compared preoperative with postoperative hearing results. An experienced radiologist extracted data about stapes prostheses from U-HRCT images, including absolute insertion depth, relative insertion depth, angle between the prosthesis and incus, angle between the prosthesis and footplate, and the relative position of the hook clamped onto the long process of incus. We analyzed potential relationships between the imaging data and postoperative hearing outcomes.

**Results:**

We obtained satisfactory hearing results from 129 ears postoperatively. 119 ears (92.2%) presented Air-Bone Gaps (ABG) in the 0–20 dB range. We observed statistically significant differences between mean pre- and post-operative ABG at different frequencies (0.5, 1, 2, and 4 kHz). The best results occurred at 2 kHz (98.5%). We found that the observed values for prosthesis insertion depth (mean 0.6 mm, relative depth: mean 23%) and mean angle between the prosthesis and incus (mean 91.2 °) were safe and effective. We also found that the mean angle between prosthesis and footplate was related to postoperative ABG (p = 0.049). The hook position on the long process of incus (relative distance of 10.5%) is also a crucial factor in determining postoperative hearing results (p = 0.726).

**Conclusion:**

Prosthesis position and postoperative hearing outcomes are related. Postoperative imaging evaluation is especially important for patients who did not benefit substantially from stapes surgery.

**Level of evidence:**

Level 3.

## Highlights

• Comparison between mean pre- and post-operative ABG.
• The relationship between prosthesis position and post-operative ABG.
• The advantages of Ultra-High Resolution Computed Tomography (U-HRCT).

## Introduction

Otosclerosis (OS) is a prevalent cause of conductive hearing loss. It has a prevalence of 0.3%–0.4% in Caucasians and 0.03%–0.1% in the African and Asian populations,^1^ and the female-to-male ratio is 2:1.^2^ Its pathological feature is primary spongiform degeneration of the labyrinth bone, which is characterized by abnormal bone resorption and bone accumulation in the temporal bone.^3^ The diagnosis of OS is then confirmed during surgery when stapes fixation is observed. Hearing improvement is the main objective for both patients and surgeons. Stapedotomy is currently the most popular surgery, and is also the main procedure adopted in our clinical center.^4^

In general, hearing outcomes after stapes surgery are promising, in both short-term and long-term follow-up periods. The postoperative Air-Bone Gap (ABG) less than 10 dBHL is defined as perfect result. Nevertheless, not all the patients can meet this criterion. Previous studies have investigated a variety of potential predictors for hearing outcomes after stapes surgery, such as preoperative ABG, lesion invasion degree, surgical procedure, surgical skills of the surgeon, prosthesis type, age, and gender.^5^, ^6^, ^7^ Among these factors, choice of prosthesis type and insertion characteristics are typical concerns of surgeons because of their potential for improvement and better outcomes, but also their potential complications, such as vertigo and sensorineural hearing loss.^8^ There is currently no consensus on the relevance of these factors. Previous studies were limited by the relatively low sensitivity of High-Resolution CT (HRCT) in estimating several parameters, such as accurate measurement of the thickness of the footplate, protrusion depth of the prothesis, and angle of the prothesis-footplate. Accurate estimation of these parameters requires images of much higher resolution.

This study builds upon previous research by our group on the use of Ultra-High Resolution CT (U-HRCT) for stapes measurements and OS diagnosis,^9^^,^^10^ with a specific focus on outcome evaluation of stapes surgery. We analyze regular audiological and clinical features of OS patients before and after surgery. To identify the impact of prosthesis on hearing outcomes we study potential relationships between the position of the stapes and hearing outcomes via measurement of multiple dimensions (including both lengths and angles) from U-HRCT images. These research findings can guide our surgical procedures to achieve the optimal surgical outcomes.

## Methods

### Patient selection and data collection

We recruited 41 OS patients (50 ears) who underwent stapes surgeries between January 2020 and June 2023. Surgery was performed by a single surgeon at the Department of Otolaryngology Head and Neck Surgery of Friendship Hospital. The study protocol was approved by the institutional Ethics Committee and the approval number is 2023-P2-274-01.

The diagnosis of OS is based on clinical manifestations and audiological findings, and confirmed by surgery. Although CT examination is not essential for the diagnosis of OS, but it is particularly important for differential diagnosis. Through CT examination, other causes of conductive hearing loss can be excluded, such as superior semicircular canal dehiscence syndrome and tympanosclerosis. In our center, U-HRCT was used for the preoperative imaging evaluation based on the superior imaging quality and one-third of the radiation dose compared with Multi-Slice CT (MSCT) (82.99 μSv vs. 252.56 μSv) when the patients present with corresponding symptoms and signs for differential diagnosis.^9^ Meanwhile, the data of pure-tone audiometry, acoustic immittance measurement and Gelle’s test were collected. The audiological evaluation for the diagnosis of OS mainly incompasses pure-tone audiometry, acoustic immittance measurement and Gelle’s test.

We adopted the following inclusion criteria: the diagnosis of OS was confirmed by surgery, and stapes surgery was performed under general anesthesia. Patients who underwent revision stapes surgery or affected by other middle-ear disorders were excluded.

We extracted the following information from patient records: demographic and clinical data, including sex, age, family history, laterality, duration of hearing loss, pre- and postoperative audiometry results (collected 3-months to 1-year after surgery), postoperative Ultra-High-Resolution CT (U-HRCT) if the patient had them, punching tools (whether laser-aided or not), and complications. Each ear was separately enrolled if both ears of a given patient were eligible.

### Surgical procedure

All operations were performed by the same surgeon. The surgeon made an endaural skin incision, elevated the tympanomeatal flap, curetted the posterosuperior bony overhang, and exposed the stapes, incudostapedial joint, tympanic segment of facial nerve, and the base of the pyramidal eminence. The stapedial tendon and both crura of the stapes were cut using laser or scissors, and the stapes superstructure was removed. During the stapedotomy procedure, fenestration was made at the posterior half of the footplate using laser or manual perforators. A Stapes Prothesis Titanium (SPIGGLE & THEIS, 0.4 mm shaft diameter) was positioned into the hole, with the other end encircling the long process of the incus and being crimped. The surgeon verified the mobility of the ossicular chain, sealed the fenestration with fat, and sutured the incision.

### Surgical outcome evaluation

We conducted pre-and postoperative pure-tone audiometry measurements, which inclued Bone Conduction (BC) and Air Conduction (AC) at frequencies of 500 Hz, 1 kHz, 2 kHz, and 4 kHz. Both the average hearing level and the ABG were calculated. We then compared the preoperative with postoperative hearing results.

### Postoperative U-HRCT evaluation

After the surgery, we advised the patients to undergo the postoperative imaging with the U-HRCT. This was also based on the need to confirm the prosthesis position and for potential comparison in long-term follow-up, especially for the patients who underwent aggravated vertigo, dizziness, newly emerging annoying tinnitus or less-than-ideal postoperative audiological results. Finally, we obtained the postoperative imaging data from 41 patients (50 ears).

Volumetric axial images were recorded unilaterally for each ear using a U-HRCT scanner (Ultra 3D, LargeV Inc., Beijing, China) using the following parameters: 100 kVp; 120–200 mAs; voxel size: 0.1 × 0.1 × 0.1 mm; field-of-view: 65 × 65 mm; exposure time: 40 s.^9^ The same radiologist examined all CTs.

**Fig. 1:**
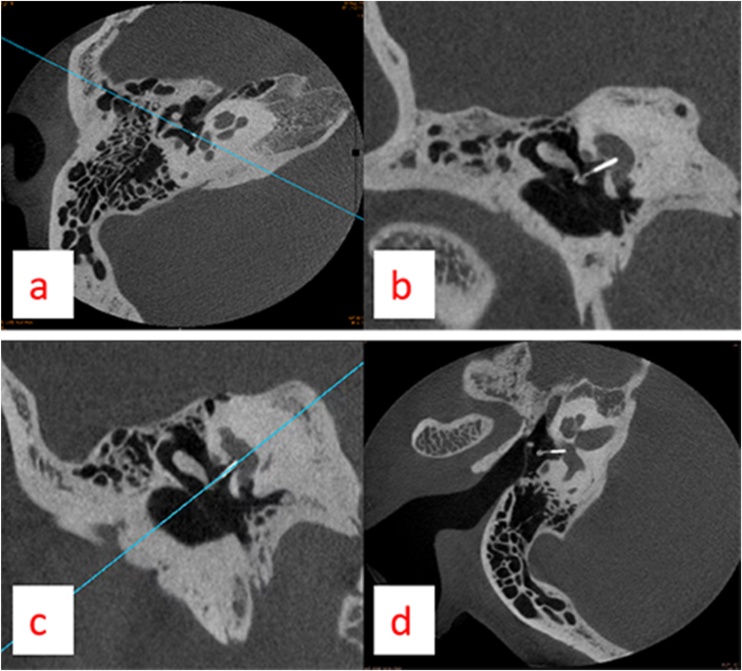
Before measuring the position of the prosthesis, we performed Multiple Planar Reconstruction (MPR) of the prosthesis. First, on the original image (a) we performed an oblique coronal plane reconstruction along the long axis of the prosthesis, which is the coronal plane of the prosthesis (b). Then, performed MPR along the long axis of the prosthesis on the coronal plane image of the prosthesis (c), which is the transverse plane of the prosthesis (d).

Before measuring the position of the prosthesis, we performed Multiple Planar Reconstruction (MPR) of the prosthesis (Fig. 1). First, on the original image (a), we performed an oblique coronal plane reconstruction along the long axis of the prosthesis, obtaining the coronal plane of the prosthesis (b). Then, we performed MPR along the long axis of the prosthesis on the coronal plane image of the prosthesis (c), thus getting the transverse plane of the prosthesis (d).

**Fig. 2:**
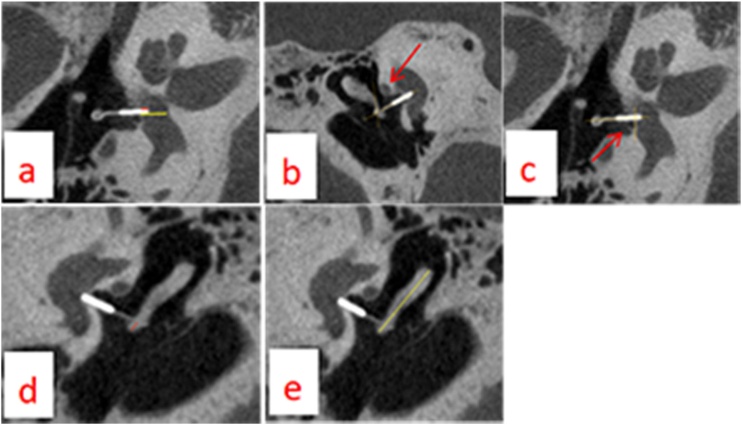
Images of the U-HRCT from OS patients undergone stapes surgery. Measurement of stapes prothesis position from (a to c). (a) The red line and yellow line represent absolute insertion depth and vestibular depth. The relative insertion depth is the ratio of both. (b) The two crossed yellow lines parallel to the long crus of incus and prothesis respectively, and red arrow shows the angle between them. (c) The two crossed yellow lines parallel to the prothesis and the footplate respectively, and red arrow shows the angle between them (d and e). The red line shows the distance between the hook and the end of incus, the yellow ratio of both line shows the length of incus. The relative distance is the.

We measured six prosthesis-specific parameters from the U-HRCT images of 41 patients (50 ears): absolute insertion depth, relative insertion depth (absolute depth/vestibular depth), the angle between the prosthesis and the incus, the angle between the prosthesis and the footplate, the absolute distance between the hook and the end of the incus, and relative distance (the absolute distance between the hook and the end of the incus divided by the length of the incus). The CT images were evaluated by the same experienced radiologist. The measurement methods adopted for imaging evaluation are shown in Fig. 2.

### Statistical analyses

All statistical tests were carried out using SPSS 27.0. We employed two related samples test (Wilcoxon one-sample test) to compare the average values between preoperative and postoperative hearing outcomes. Additionally, we utilized one-way ANOVA analysis to identify factors influencing postoperative hearing results. A p-value of less than 0.05 was regarded as statistically significant.

## Results

The demographic and clinical characteristics of 41 patients (50 ears) are presented in Table 1. In total, we included 8 males (19.5%) and 33 females (80.5%), with an average age of 42.56 years (ranging from 18 to 73 years) at the time of surgery. One patient (2%) had a relevant family history. The average duration of hearing loss at the time of surgery was 5.93 years (ranging from 0.5 to 30 years). Unilateral OS and bilateral OS were observed in 7 (14%) and 43 (86%) patients, respectively. Laser-assisted surgery was performed on 43 (86%) patients.

**Table 1:** Demographics and clinical characteristics of 50 ears (41 patients).

Characteristic	n (%)
Age at operation (years)	42.56 ± 12.20
Gender (cases)	
Male	8 (19.5%)
Female	33 (80.5%)
Family history	
Yes	1 (2%)
No	49 (98%)
Duration of hearing loss (years)	5.93 ± 5.26
Laterality (cases)	
Unilateral	7 (14%)
Bilateral	43 (86%)
Operating ear	
Left	28 (56%)
Right	22 (44%)
Laser use or not	
Yes	43 (86%)
No	7 (14%)
Preoperative tinnitus	
Yes	38 (76%)
No	12 (24%)
Preoperative vertigo	
Yes	5 (10%)
No	45 (90%)

**Fig. 3:**
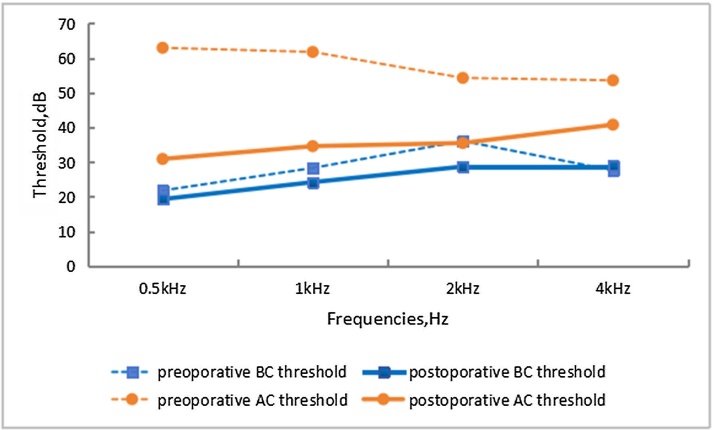
Mean BC and AC threshold at preoperative and postoperative audiometry for 50 ears (41 patients).

We obtained pre-and post-operative hearing results from 41 patients (50 ears). The mean AC and BC thresholds (500 Hz, 1 kHz, 2 kHz, and 4 kHz) before and after surgery are presented in Fig. 3. For the four different frequencies, the average AC threshold improved respectively: from 63.0 to 30.9 dB (p < 0.001), from 61.8 to 34.6 dB (p < 0.001), from 54.3 to 35.5 dB (p < 0.001), and from 53.6 to 40.8 dB (p < 0.001). The mean AC improved from 58.2 to 35.5 dB (p < 0.001). No deterioration of the BC threshold was measured at the four frequencies. The ABG closure values for the four frequencies after surgery were 11.6 dB, 10.4 dB, 6.8 dB, and 11.9 dB, with an average ABG value of 10.2 dB. In our study, 64% of the ears (32/50) achieved successful results with average ABG values below 10 dB, while 26% of the ears (13/50) had satisfactory results with average ABG values between 10 dB and 20 dB, and 10% of the ears (5/50) had average ABG values above 20 dB post-operatively. We analyzed the ABG results across different frequencies (Table 2) and found that the best results occurred at 2 kHz (50 ears, 100%), followed by 1 kHz (47 ears, 94%), 500 Hz (45 ears, 90%) and 4 kHz (42 ears, 84%).

**Table 2:** Postoperative ABG analysis in average value and in different frequencies for the 50 ears.

ABG	500 Hz	1 kHz	2 kHz	4 kHz	Average	p
ABG ≤ 10dB	33 (66%)	33 (66%)	40 (80%)	27 (54%)	32 (64%)	<0.001
10 < ABG ≤ 20 dB	12 (24%)	14 (28%)	10 (20%)	15 (30%)	13 (26%)	
ABG > 20 dB HL	5 (10%)	3 (6%)	0 (0%)	8 (16%)	5 (10%)

**Fig. 4:**
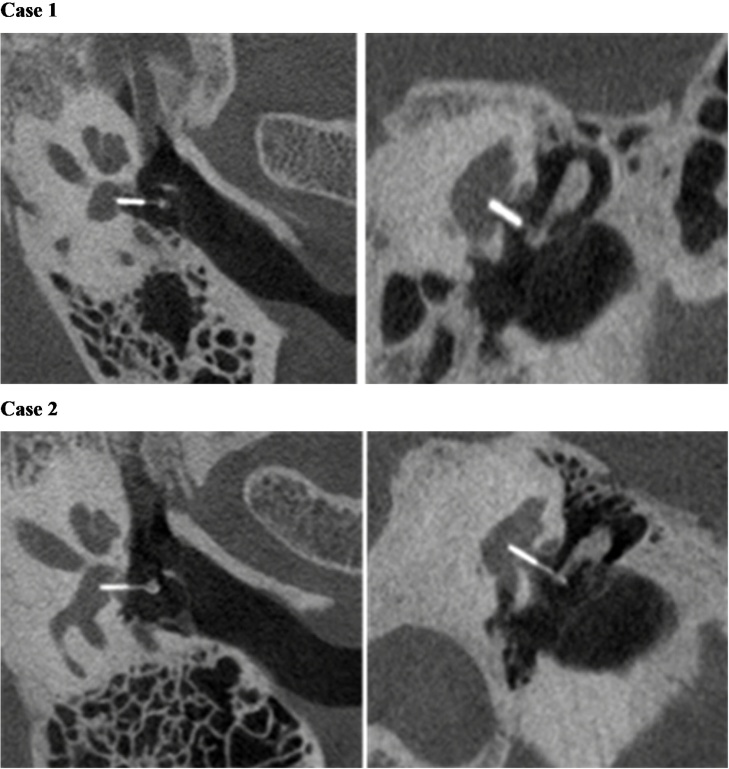
Postoperative radiologic images of successful case (Case 1) and unsuccessful case (Case 2). (Case 1) The ABG value was 5 dB after operation. The left image is the transverse plane of the prosthesis, the right image is the coronal plane of the prosthesis. The absolute insertion depth into the vestibule was 0.0 mm, the vestibulum depth was 2.7 mm, the ratio between absolute insertion depth and vestibulum depth was 0%; the angle between the prosthesis and the incus was 88.4 °, the angle between the prosthesis and the footplate was 103.1; the distance between the hook and the end of the incus was 0.6mm, the relative distance between the hook and the end of the incus was 9.7%. (Case 2) The ABG value was 23 dB after operation. The left image is the transverse plane of the prosthesis, the right image is the coronal plane of the prosthesis. The absolute insertion depth into the vestibule was 0.9 mm, the vestibulum depth was 3 mm, the ratio between absolute insertion depth and vestibulum depth was 30%; the angle between the prosthesis and the incus was 95.6 °, the angle between the prosthesis and the footplate was 112.4 °; the distance between the hook and the end of the incus was 0.3 mm, the relative distance between the hook and the end of the incus was 4.8%.

We obtained U-HRCT imaging data from 50 ears with the following postoperative ABG distribution: 32 ears presented ABG values below 10 dB, 13 ears between 10 dB and 20 dB, and five ears above 20 dB. Measurement data are presented in Table 3. Table 4 summarizes the results of one-way ANOVA analysis applied to the measurement values of the stapes prosthesis position for different postoperative ABG. The mean absolute insertion depth into the vestibule was 0.7 mm (SD = 0.4 mm), 0.5 mm (SD = 0.2 mm), and 0.7 mm (SD = 0.4 mm) for the 0–10 dB, 10–20 dB, and 20–30 dB groups, respectively (p = 0.448). The mean ratio between absolute insertion depth and vestibulum depth was 24% (SD = 16%), 20% (SD = 9%), and 25% (SD = 14%) for the 0–10 dB, 10–20 dB, and 20–30 dB groups, respectively (p = 0.630). The mean angle between the prosthesis and the incus was 91.6 ° (SD = 9.3 °), 89.6 ° (SD = 7.5 °), and 92.9 ° (SD = 7.0 °) for the 0–10 dB, 10–20 dB, and 20–30 dB groups, respectively (p = 0.706). The mean angle between the prosthesis and the footplate was 97.7 ° (SD = 9.4 °), 88.4 ° (SD = 18 °), and 101.3 ° (SD = 11.6 °) for the 0–10 dB, 10–20 dB, and 20–30 dB groups, respectively (p = 0.049). The mean relative distance between the hook and the end of the incus was 10.5% (SD = 2.4%), 11.2% (SD = 3.5%), and 11.3% (SD = 4.8%) for the 0–10 dB, 10–20 dB, and 20–30 dB groups, respectively (p = 0.726).

**Table 3:** The measurement values of stapes prosthesis position in otosclerosis patients (50 ears).

Factor	Minimum ‒ Maximum	Mean ± SD
Absolute insertion depth (mm)	0.0 ‒ 1.7	0.6 ± 0.4
Vestibular depth (mm)	1.6 ‒ 3.9	2.8 ± 0.3
Relative insertion depth (%) (Absolute depth/vestibular depth)	0 ‒ 55	23.3 ± 13
Angle between the prosthesis and incus (°)	73 ‒ 113	91 ± 8.5
Angle between the prosthesis and footplate (°)	43 ‒ 121	95 ± 12.9
Absolute distance between hook and end of incus (mm)	0.3 ‒ 1.1	0.7 ± 0.2
The length of incus	6.0 ‒ 7.9	6.6 ± 0.4
Relative distance^a^ (%)	5 ‒ 17	11 ± 3

a Absolute distance between hook and end of incus/the length of incus.

**Table 4:** The measurement values of stapes prosthesis position for different postoperative ABG (dB HL, 50 ears).

Factor	0 ≤ ABG ≤ 10	10 < ABG ≤ 20	20 < ABG ≤ 30	F	p
Absolute insertion depth (mm)	0.7 ± 0.4	0.5 ± 0.2	0.7 ± 0.4	0.816	0.448
Relative insertion depth (%) (Absolute depth/vestibular depth)	24 ± 16	20 ± 9	25 ± 14	0.467	0.630
Angle between the prosthesis and incus (°)	91.6 ± 9.3	89.6 ± 7.5	92.9 ± 7.0	0.351	0.706
Angle between the prosthesis and footplate (°)	97.7 ± 9.4	88.4 ± 18	101.3 ± 11.6	3.213	0.049
Absolute distance between hook and end of incus (mm)	0.6 ± 0.1	0.7 ± 0.2	0.8 ± 0.3	0.782	0.463
Relative distance^a^ (%)	10.5 ± 2.4	11.2 ± 3.5	11.3 ± 4.8	0.323	0.726

a Absolute distance between hook and end of incus/the length of incus.

## Discussion

In our study, we achieved satisfactory postoperative hearing outcomes in 50 ears. Moreover, we demonstrated more precise positional measurements of stapes prostheses when estimated from U-HRCT images. This enabled us to analyze relevant absolute/relative depths and angles for the evaluation of hearing improvement. We believe that the prosthesis insertion depth (mean 0.6 mm, relative depth ranging from 0 to 55%, mean 23%) and the mean angle between the prosthesis and the incus (mean 91.2 °) observed in our study represent safe and effective reference choices for the successful implementation of the surgical procedure. We measured a suggestive correlation between the prosthesis-footplate angle and postoperative ABG.

Odat and colleagues^6^ analyzed the hearing results of 51 patients (58 ears) after stapedotomy. They found that a postoperative ABG of 10 dB or lower was achieved in 44 cases, with a success rate of 75.9%. However, Wedel and collaborators^11^ reported a lower success rate of 59% in 93 cases. These differences in reported success rates may be attributable to the different sample sizes adopted. In a study by Lucidi,^12^ 70 patients (107 ears) were followed up for 22 years after stapes surgery, they found no statistically significant differences between early and late postoperative AC-PTA, nor differences in BC-PTA. In the present study, we analyzed hearing results of 43 patients (50 ears), and we found significant differences in the mean preoperative and postoperative ABG at different frequencies (0.5, 1, 2, and 4 kHz). A mean ABG of 10 dB or lower was achieved in 64% of the cases, while 90% achieved ABG values of 20 dB or lower.

There are many different preoperative, intraoperative, and postoperative factors presumed to influence final hearing outcomes. Most studies have reached a consensus that preoperative ABG was an independent prognostic factor affecting hearing outcomes after stapes surgery.^11^^,^^13^^,^^14^ Relevant intraoperative and postoperative factors include surgery technique, absence/presence of laser assistance, prosthesis choice, prosthesis insertion position, possible multiple lesion sites, and prosthesis subluxation. In this study, we aimed to focus on objective prosthesis-specific factors. In previous studies, CT has been used to evaluate prothesis insertion depth and position, and also for the exploration of possible reasons underlying poor results or complications. However, the results of these studies may have been affected by the relatively poor image resolution of delicate structures like the ossicular chain.^15^ In our study, we relied on U-HRCT to acquire the postoperative imaging parameters including prosthesis depth and angles. Compared with MSCT, U-HRCT can provide more accurate estimation of stapes prosthesis parameters.^10^ The image quality of U-HRCT is significantly superior to that of MSCT. In addition to its lower radiation dose (the effective dose of U-HRCT was one-third of that of MSCT: 82.99 μSv vs. 252.56 μSv), U-HRCT is patient-friendly and a very good postoperative evaluation tool for middle ear surgery.^9^

The choice of the stapes prosthesis length depends on the distance between the incus and the footplate, a factor considered crucial as it may impact postoperative outcomes. Husain and colleagues^16^ utilized prostheses of different lengths, ranging from 3.75 mm to 4.75 mm (median: 4.25 mm), in 227 OS patients. They observed the greatest improvement in postoperative ABG in the 4.25 mm group. However, this study did not detail the insertion depth into the vestibule, a potentially relevant factor when compared with the absolute piston length, given the varying sizes of the middle ear cavity. Excessively deep intravestibular insertion is thought to cause postoperative vertigo or sensorineural hearing loss, which seriously affect the life of patients.^17^ Conversely, if the stapes prosthesis is inserted too shallow, it may slip into the tympanic cavity when sneezing or undergoing other middle ear pressure changes, resulting in a recurrent conductive hearing loss.^17^ Thus, proper choice of insertion depth for the piston is a key factor in stapes surgery. Nevertheless, the optimal insertion depth remains elusive. In a 1991 histologic study of human temporal bones, Pauw and colleagues^18^ reported a “safe” intravestibular prosthesis depth range of 0.5‒1.7 mm. Gil et al.^19^ found that the prosthesis insertion depth varied from 0.2 to 1.6 mm (mean: 0.74 mm), and the ratio between insertion depth and vestibule depth ranged from 8% to 59% (mean: 26.6%). In these 39 cases, they found no relationship between insertion depth and postoperative BC. Fang et al.^15^ observed larger values for prosthesis depth (ranging from 0.8 to 2.5 mm with relative depth from 22% to 88%) than those reported by Gil. Variations in image quality, slice thickness, and interval may account for the differences in reported prosthesis depth. In our study, the intravestibular prosthesis depth estimated from U-HRCT ranged from 0 to 1.7 mm (mean: 0.6 mm), with a relative depth of 0%–55% (mean: 23%). This rang is lower than those reported by Gil and Fang but consistent with that of Pauw. When measuring the mean relative insertion depth, the values for the 0–10 dB, 10–20 dB, and 20–30 dB groups were 24%, 20%, and 25% respectively. There was no statistical difference in postoperative ABG among the three groups. Given the satisfactory postoperative result in our study (92.2% with ABG ≤ 20 dB), we believe that the prosthesis insertion depth observed in this study (mean: 0.6 mm, mean relative depth: 23%) is safe and effective. No patient in our study experienced persistent vertigo or Sensorineural Hearing Loss (SNHL) after surgery, likely due to all procedures being performed by the same skilled surgeon, who avoided incorrect insertion depth choices. More cases with accurate postoperative measurements will be needed in future research.

In stapes surgery, the attachment of the prosthesis to the long process of the incus probably plays a crucial role in hearing improvement due to the mechanics of the ossicular chain. Moreover, this factor is associated with the occurrence of late-stage complications, such as incus erosion and necrosis. It is believed that the ideal angle between the prosthesis and the long process of the incus is 90 °, and the prosthesis should be fixed to the long process of the incus with appropriate strength.^20^ However, there are no studies investigating the correlation between these factors and hearing outcomes. In the present study, we measured a mean angle between the prosthesis and the incus of 91.2 ° (ranging from 73.5 ° to 113.7 °) in 50 ears. For the 0–10 dB, 10–20 dB, and 20–30 dB groups, the mean angles were 91.6 ° (SD = 9.3 °), 89.6 ° (SD = 7.5 °), and 92.9 ° (SD = 7.0 °) respectively, with no statistically significant difference among the groups. Our results are consistent with previous studies suggesting that 90 ° is an appropriate angle for favorable postoperative results. In this study, we also measured the position of the hook on the long process of the incus. The mean relative distances between the hook and the end of the incus were 10.5% (SD = 2.4%), 11.2% (SD = 3.5%), and 11.3% (SD = 4.8%) in the 0–10 dB, 10–20 dB, and 20–30 dB groups respectively. Currently, there is no relevant research providing guidance for surgeons regarding this parameter. If the relative distance is too short, the hook may easily slip away from the incus; if it is too long, the stapes prosthesis may not produce effective vibration, thereby affecting postoperative hearing outcomes. Is the angle between prosthesis and footplate related to postoperative hearing outcomes? There is no relevant study in the literature. In the present study, we found that the mean angle between prosthesis and footplate was related to postoperative ABG. The mean angles were 97.7 ° (SD = 9.4 °), 88.4 ° (SD = 18 °), and 101.3 ° (SD = 11.6 °) in the 0–10 dB, 10–20 dB, and 20–30 dB groups respectively (p = 0.049). However, after pairwise comparison among the three groups, we found no significant statistical difference. Further research is required to clarify this issue. In our study, this question remains unresolved, possibly due to the limited number of cases available.

## Conclusion

The position of the prosthesis is related to postoperative hearing outcomes. A proper prosthesis position can result in favorable postoperative hearing outcomes and reduce the incidence of postoperative complications, such as vertigo, incus erosion and so on. Not all OS patients require postoperative imaging evaluation. However, postoperative imaging evaluation is especially important for patients who did not benefit substantially from stapes surgery. Limitations of this study: The number of patients who voluntarily underwent U-HRCT examination after surgery was relatively small, which may have led to sample selection bias.Therefore, further research is needed.

## ORCID ID

Chen Yang: 0000-0002-6412-2459

Heyu Ding: 0000-0003-1519-6141

Ting Zhang: 0009-0008-6769-1834

Pengfei Zhao: 0000-0002-9210-6544

Zhenchang Wang: 0000-0001-8190-6469

Shusheng Gong: 0000-0001-6847-3195

## Funding

The 10.13039/501100001809National Natural Science Foundation of China (grant number 82071054).

## Declaration of competing interest

The authors declare no conflicts of interest.
